# 17β-estradiol suppresses lipopolysaccharide-induced acute lung injury through PI3K/Akt/SGK1 mediated up-regulation of epithelial sodium channel (ENaC) *in vivo* and *in vitro*

**DOI:** 10.1186/s12931-014-0159-1

**Published:** 2014-12-31

**Authors:** Di Qi, Jing He, Daoxin Wang, Wang Deng, Yan Zhao, Yuan Ye, Longhua Feng

**Affiliations:** Department of Respiratory Medicine, Second Affiliated Hospital of Chongqing Medical University, 76 Linjiang Road, Yuzhong District, Chongqing, 400010 China

**Keywords:** Acute lung injury (ALI), 17β-estradiol, Epithelial sodium channel (ENaC), Phosphoinositide 3-kinase (PI3K), Akt, Serum and glucocorticoid-induced kinanse-1 (SGK1)

## Abstract

**Background:**

17β-estradiol can suppress acute lung injury (ALI) and regulate alveolar epithelial sodium channel (ENaC). However the relationship between these two functions remains unclear. This study is conducted to assess the role of ENaC and the PI3K/Akt/SGK1 signaling pathway in 17β-estradiol therapy in attenuating LPS-induced ALI.

**Methods:**

ALI was induced in C57BL/J male mice by intratracheal administration of lipopolysaccharide (LPS). Concurrent with LPS administration, 17β-estradiol or sterile saline was administered to ALI model with or without the phosphoinositide 3-kinase (PI3K) inhibitor wortmannin. The lung histological changes, inflammatory mediators in bronchoalveolar lavage fluid (BALF), wet/dry weight ratio (W/D) and alveolar fluid clearance (AFC) were measured 4 hours after LPS challenge *in vivo*. For *in vitro* studies, LPS-challenged MLE-12 cells were pre-incubated with or without wortmannin for 30 minutes prior to 17β-estradiol treatment. Expression of ENaC subunits was assessed by reverse transcriptase PCR, western blot, cell surface biotinylation, and immunohistochemistry. The levels of phosphorylated Akt and SGK1 in lung tissue and lung cell lines were investigated by western blot.

**Results:**

17β-estradiol suppressed LPS-mediated ALI in mice by diminishing inflammatory mediators and enhancing AFC. 17β-estradiol promoted the expression and surface abundance of α-ENaC, and increased the levels of phosphorylated-Akt and phosphorylated-SGK1 following LPS challenge. This induction was abolished by the PI3K inhibitor wortmannin *in vivo* and *in vitro*.

**Conclusion:**

17β-estradiol attenuates LPS-induced ALI not only by repressing inflammation, but also by reducing pulmonary edema via elevation of α-ENaC expression and membrane abundance. These effects were mediated, at least partially, via activation of the PI3K/Akt/SGK1 signaling pathway.

## Introduction

Acute Respiratory Distress Syndrome (ARDS), the severe stage of Acute Lung Injury (ALI), is a devastating condition with a 30-60% mortality rate [1-3]. Several clinical studies have indicated that females are more resistant to ARDS, with lower morbidity and improved outcomes compared to male patients [3-8]. Moreover, experimental studies in animal models suggest that 17β-estradiol, the primary circulating estrogen in humans and animals, can have therapeutic effects on ALI through a variety of mechanisms, including modulation the inflammatory response [9-14].

In addition to inflammation, ALI/ARDS induces extensive capillary damage, leading to non-cardiogenic pulmonary edema. Therefore, the rate of alveolar fluid clearance (AFC) is a crucial prognostic factor for ALI/ARDS patients. Specifically, a reduced AFC rate is associated with higher mortality in ARDS patients [15,16]. AFC is mediated by ion transporters, including the alveolar epithelial sodium channel (ENaC). ENaC is a heteromultimeric protein composed of α, β and γ subunits. This transporter is essential for the transepithelial absorption of sodium and fluid from alveolar spaces [17,18].

Recent studies have suggested a role for female sex hormones (estrogen and progesterone) in the physiology of alveolar ENaC. A recent single center study indicates that women with ALI/ARDS-associated lung edema have significantly higher rates of AFC compared to men [19]. Furthermore, ENaC mRNA levels are higher in female rats compared to males [20]. In female rats, membrane α-ENaC abundance is highest during the proestrus stage of the estrus cycle, coinciding with maximal 17β-estradiol levels [21]. Pharmacological prenatal deprivation of 17β-estradiol and progesterone decreases amiloride-sensitive AFC in newborn piglets, suggesting a role for ENaC in controlling AFC [22]. Moreover, co-administration of 17β-estradiol and progesterone can increase sodium transport in alveolar epithelial cells by enhancing the expression and activity of ENaC [23]. Taken together, these studies suggest that female sex hormones can promote ion transport and increase AFC by regulating alveolar ENaC.

17β-estradiol exerts many of its biological functions through regulation of gene transcription. However, 17β-estradiol can also act through non-genomic mechanisms to rapidly activate signaling pathways and modulate protein expression, function and distribution [24,25]. For example, 17β-estradiol regulates phosphoinositide-3 kinase (PI3K) and its direct downstream target protein kinase B (PKB) (also known as Akt) to control inflammation, proliferation and immunity [26-29]. This pathway provides a negative feedback mechanism for sepsis, inflammation and ischemia/reperfusion injury [30-32]. Moreover, the PI3K/Akt signaling pathway can activate serum and glucocorticoid-induced kinanse-1 (SGK1), a kinase that can promote ENaC expression and activity [33-35]. This could provide a potential mechanism by which PI3K could regulate sepsis, inflammation, and ischemia/reperfusion injury, however this has yet to be demonstrated conclusively.

Consistent with these findings, activation of the PI3K/Akt by growth factors, hormones, or cytokines exerts protective effects in animal models of ALI [36-39]. Furthermore, estrogen is known to activate the PI3K/Akt signaling pathway, contributing to the attenuation of lung injury induced by trauma-hemorrhage and acute pancreatitis [40,41]. Taken together, these findings suggest that PI3K-dependent activation of ENaC by 17β-estradiol may contribute to the gender dimorphism observed in ALI/ARDS patients. However, this hypothesis has not been adequately tested in experimental models.

Our present study aimed to confirm the effects of 17β-estradiol on LPS-induced ALI, a classical model of Gram-negative bacteria induced ALI. We specifically investigated the effects of 17β-estradiol on ALI-associated pulmonary edema, ENaC expression, and the role of the PI3K/Akt/SGK1 signaling pathway in these effects.

## Materials and methods

### Drugs and regants

Lipopolysaccharide (E. coli LPS serotype 0111: B4), 17β-estradiol, wortmannin (PI3K inhibitor), Evans blue, and sodium pentobarbital were purchased from Sigma (St Louis, MO, USA). Rabbit anti-α-ENaC, β-ENaC, γ-ENaC, β-actin, pan-cadherin, rabbit anti- phospho-Akt (Ser308, Ser473) and anti-total Akt antibodies were obtained from Abcam (Cambridge, MA, USA). Rabbit anti-phospho-SGK1 (Ser422, Thr256) and anti-total SGK1 antibodies were purchased from Santa Cruz Biotechnology (Santa Cruz, CA, USA). RNAiso plus, the PrimeScript RT Reagent Kit, and Premix Taq version 2.0 were purchased from TaKaRa Biotechnology (Dalian, China).

### Animal preparation

All animal experiments were carried out on ten-week-old male BALB/c mice (19–25 g) of SPF grade (Department of Laboratory Animal Center, Chongqing Medical University) following a minimum facility acclimatization period of 7 days. All animals were housed in an air-conditioned room under a 12 hour (h) dark/light cycle and were granted free access to water and food. All experimental protocols involving animals were approved by the Ethics Committee of the Second Affiliated Hospital of Chongqing Medical University and implemented in accordance with the instructions of National Institutes of Health Guild for the Care and Use of Laboratory Animals.

### LPS-induced ALI model in mice

Forty healthy male BALB/c mice were randomly divided into four groups (n =10): control group, LPS group, LPS + 17β-estradiol group (E_2_ group), LPS + 17β-estradiol + wortmannin group (wortmannin group). Mice in each group were anesthetized by intraperitoneal administration of sodium pentobarbital (50 mg/kg). Mice were intratracheally instilled with 5 mg/kg LPS in 50 μL sterile saline, or sterile saline alone (control group) with an indwelling vein needle. At the time of LPS exposure, 1 mg/kg 17β-estradiol in 100% ethanol was administered intravenously to mice in the E_2_ and wortmannin groups, while the control and LPS groups received an equal volume of sterile saline. Mice in the wortmannin group received 16 μg/kg wortmannin intravenously 30 minutes prior to LPS injection, while other groups were given sterile saline. Four hours after LPS injection, the mice were sacrificed and bronchoalveolar lavage fluid (BALF) and lung tissues were collected.

### Cell counts, protein levels, TNF-α, IL-6, myeloperoxidase (MOP) assay in BALF

Mice were anesthetized with pentobarbitone (50 mg/kg I.P.) and tracheal intubations were performed. Then 1 mL preheated sterile saline was administered into the lung and extracted three times via a tracheal catheter. BALF was collected and kept on ice and centrifuged at 1200 × g for 10 min at 4°C to remove cell debris. The pellets were resuspended in 50 μL PBS and stained with Wright-Geimsa (KeyGen Biotech Co., Nanjing, China). Total cells and neutrophils were counted with hemocytometer in a double-blind manner. Protein levels in the BALF supernatants were determined using bicinchoninic acid protein assay (BCA) kit (KeyGen Biotech Co., Nanjing, China). An aliquot of BALF supernatant were used to assay the TNF-α, IL-6 levels using the respective ELISA kits (R&D, Minneapolis, MN, USA). MPO activity was measured with MPO assay kit (Nanjing Jiancheng Bioengineering Institute, Nanjing, China). All assays were conducted under the manufacturer’s instructions.

### Lung wet/dry weight ratio

The lungs wet to dry weight ratio (W/D ratio) was measured to evaluate pulmonary edema. After being weighed, the right lower lungs were dehydrated in an oven at 80°C for 24 hours. Then the dry weight was measured again to calculate W/D ratio.

### Alveolar fluid clearance (AFC)

The AFC rate was estimated by determining the alveolar Evans Blue-labeled albumin concentrations. One mL of warm sterile saline (5 ml/kg) containing Evans Blue-dyed 5% bovine albumin (0.15 mg/ml) was injected into the lung with 2 ml oxygen to facilitate distribution. Lungs were ventilated with 100% oxygen at an airway pressure of 7 cm H_2_O in a humidified incubator at 37°C for 1 h. Alveolar fluid was aspirated and labeled albumin was measured by a spectrophotometer at 620 nm. AFC was calculated as following formula:

$$ \mathrm{A}\mathrm{F}\mathrm{C} = \left[\left(\mathrm{V}\mathrm{i}\ \hbox{--}\ \left.\mathrm{V}\mathrm{f}\right)/\mathrm{V}\mathrm{i}\right)\right]\times 100\%\ \mathrm{V}\mathrm{f} = \left(\mathrm{V}\mathrm{i}\times \mathrm{E}\mathrm{i}\right)/\mathrm{E}\mathrm{f} $$

V represents the volume of injected albumin solution (i) and final alveolar fluid (f), and E represents the injected (i) and final (f) concentrations of Evans Blue-labeled 5% albumin solution

### H&E staining and lung histology evaluation

For lung histological studies, the isolated left lung in each group was fixed in 4% paraformaldehyde, embedded in paraffin wax, cut into 5 mm sections, and stained with hematoxylin and eosin (H&E). The lung injury score was calculated by assessing the degree of inflammatory cell infiltration, hemorrhage, interstitial and alveolar edema, and the thickness of alveolar septum in five random fields in a blind manner using light microscopy. A score of 0 represented no damage; l represented mild damage; 2 represented moderate damage; 3 represented severe damage and 4 represented very severe histological damage.

### Cell culture and treatments

MLE-12 cells [American Type Culture Collection (ATCC), CRL-2110], a cell line derived from murine alveolar epithelial cells, were seeded on culture dishes in a 5% CO_2_, 95% air atmosphere in Hites medium containing 10% FBS, 0.1 mg/ml streptomycin, and 100 U/ml penicillin. The culture medium was changed every day. Once the cell reached 80% confluence, they were divided into 4 groups and serum-starved overnight. Following starvation, cells were treated as follows: (1) control group with sterile PBS, (2) LPS group with 100 ng/ml LPS, (3) 17β-estradiol group with 100 ng/ml LPS and 10nM 17β-estradiol (at the same time of LPS treatment), and (4) wortmannin group with 100 ng/ml LPS, 10nM 17β-estradiol and 30nM wortmannin (applied 30 minutes prior to 17β-estradiol treatment). Cells were incubated for 20 minutes prior to RNA and protein harvesting.

### RNA extraction and reverse transcription polymerase reaction (RT-PCR)

Total RNA was isolated from left lung or MLE-12 cells with an RNA extraction kit and was quantified by a spectrophotometer. The primer sequences used for polymerase chain reaction (PCR) amplification were as follows: α-ENaC, 5'-TACAACTCTTCCTACACTCGCCA-3'(forward), 5'-CTGGTTGAAACGACAGGTAAAGAT-3'(reverse). β-ENaC: 5'-CAATGACACCCAGTATAAGATGACC-3'(forward), 5'-CAATGAGGCACAGCACCGA-3'(reverse). γ-ENaC: 5'-CAATGAGAACGAGAAGGGAAAG-3'(forward), 5'-AAGAAGCAGGTCACCAGCAGT-3'(reverse). β-actin: 5'-CGAGCGGGCTACAGCTTC-3'(forward), 5'-GTCACGCACGATTCCCTCT-3'(reverse).

Complementary DNA was prepared using PrimeScript RT Reagent Kit. The reverse transcription reaction conditions were 37°C for 15 min and 85°C for 5 s. Polymerase chain reaction conditions were pre-denaturation at 94°C for 5 min, 35 cycles of denaturation at 94°C for 30 s, annealing at 57.8°C (α, β-ENaC), 57.0°C (γ-ENaC) for 30s, and 57.4°C (β-actin) for 40 s, and polymerization at 72°C for 60 s under the Premix Taq version 2.0 instructions. Amplified products were separated by electrophoresis on a 2.5% agarose gel containing gold view. RT-PCR products were visualized with Gel Imaging System (Bio-Rad, Hercules, Calif., USA) and analyzed with Quantity One software (Bio-Rad).

### Total protein isolation

RIPA buffer (KeyGEN Bio TECH Co., Nanjing, China) was used to extract total protein of MLE-12 cells and the left lung tissues of mice in each treatment group, following manufacture’s instructions. Briefly, 50 mg homogenized left lung tissues or cultured MLE-12 cells were lysed with 1000 μL RIPA buffer supplemented with protease and phosphatase inhibitors, incubated on ice for 10 minutes with mixing, and centrifuged at 15000 × g for 15 min at 4°C. The supernatant, containing the total soluble protein, was collected for further analysis.

### Membrane protein isolation

Mem-PER Plus Membrane Protein Extraction Kit (Thermo scientific, Weltham MA, USA) was used to extract membrane proteins of MLE-12 cells and the left lung tissues of mice in each treatment group, following manufacture’s instructions. Briefly, 50 mg left lung tissue was washed by cell wash solution, cut to pieces and homogenized in permeabilization buffer to an even suspension. Cells were scraped off, resuspended in Hites medium and centrifuged at 300 × g for 5 minutes. Cell pellet was washed with 3 mL of cell wash solution and centrifuge at 300 × g for 5 min. After complete removal of supernatant containing cytosolic extract, cells were resuspended in wash solution and centrifuged at 300 × g for 5 min. Again, supernatant was discarded and permeabilization buffer was added to cell pellet. Then the homogeneous tissue and cell suspension in permeabilization buffer was obtained and incubated at 4°C with mixing for 20 min. Again, pellet was centrifuged at 16,000 × g for 15 minutes and supernatant was discarded. The pellet was resuspended in solution buffer and incubated at 4°C with mixing for 40 min. Then suspension was centrifuged at 16,000 × g for 15 minutes at 4°C. Finally, membrane protein contained in supernatant was obtained and further analyzed by western blot.

### Western blot

An aliquot of supernatant was used to determine the protein concentration with BCA kit and mixed with 4x sodium dodecyl sulfate sample buffer. Equivalent amounts of sample were loaded into each well and separated by SDS-PAGE and electro-transferred onto Nitrocellulose membranes, blocked for 1 h with 5% dry milk/BSA and immuno-blotted with anti-α-, anti-β-, anti-γ-ENaC, anti-pan-cadherin (all 1: 1,000 dilution), β-actin (1: 8,000 dilution), anti-Akt, anti-SGK1 (all 1: 2,000 dilution), anti-phospho Akt and anti-phospho SGK1 (all 1: 1,000 dilution) primary antibodies, respectively, overnight at 4°C. Later, the membranes were washed with PBST or TBST 3 times (10 min each time) and incubated with horseradish peroxidase-conjugated anti-goat or anti-rabbit secondary antibody (1: 8,000 dilution) at 37°C for 1 h and washed 3 times again to detect bands using enhanced chemiluminescence by UVP gel imaging system (Upland, Calif., USA). The relative abundance of protein was quantified using Quantity One Software (Bio-Rad, Hercules, CA, USA).

### Immunohistochemistry

Slices of mouse left lung of each group were deparaffinized with xylene, rehydrated with a gradient of ethanol, and blocked by incubating 3% H_2_O_2_ at 37°C for 15 min. Then the slices were rinsed in PBS 3 times (10 minutes each time). Antigen retrieval was performed by immersing the slices in citric acid buffer in a microwave at 96°C for 20 min. Tissues were blocked by serum albumin in an incubator at 37°C for 30 min and the slices were incubated with primary antibodies at 4°C overnight. Tissue were washed with PBS 3 times (10 minutes each time), incubated with biotin-labeled secondary antibody at 37°C for 30 min, and then stained with DBA. Samples were counterstained with hematoxylin, dehydrated with gradient ethanol, vitrified with xylene and sealed with neutral resins. Serum was used as the primary antibody for negative control group. The number of positive cells was calculated from the average of 5 random high-power fields in a blind manner.

### Statistical analysis

Data are presented as mean ± standard error of the mean (S.E.M.). Statistical analyses performed by one-way analysis of variance (ANOVA) using SPSS 19.0 software (SPS Inc., Chicago, I11, USA). Post-hoc tests (SNK and LSD) were performed to detect significant differences between particular groups. Paired t-test was used for comparisons before and after treatment in the same group. P < 0.05 was set as the threshold value for statistical significance.

## Results

### 17β-estradiol attenuated LPS-induced lung histopathological alterations *in vivo*

**Figure 1:**
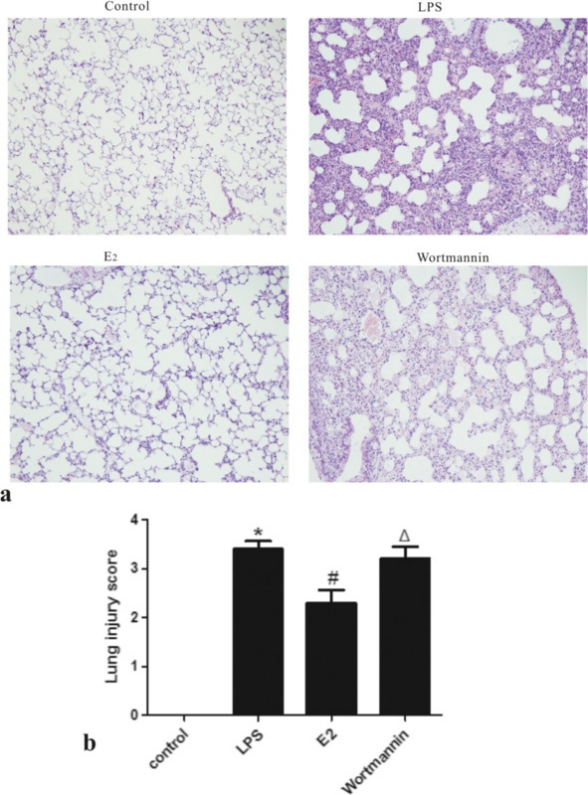
**Effects of 17β-estradiol (E**_**2**_ **) on LPS-induced lung histological alterations (a) and lung injury score (b) 4 hours after LPS challenge (H&E stain, magnification × 200).** Lung injury scores are presented as means ± S.E.M (*p < 0.05 compared with the control group, #p < 0.05 compared with the LPS group, Δp < 0.05 compared with the 17β-estradiol group).

LPS-induced lung damage was assessed by H&E staining. LPS-treated mice exhibited the typical pathological changes of ALI, including intra-alveolar and interstitial edema, hemorrhage, thickened alveolar septum, and inflammatory cell infiltration. However, all of these pathological changes were attenuated by administration of 17β-estradiol, resulting in a reduced lung injury score. In contrast, pre-treatment with wortmannin blocked the effects of 17β-estradiol (Figure 1).

### 17β-estradiol reduced IL-6, TNF-α, protein level, MPO activity and neutrophil infiltration in BALF

**Figure 2:**
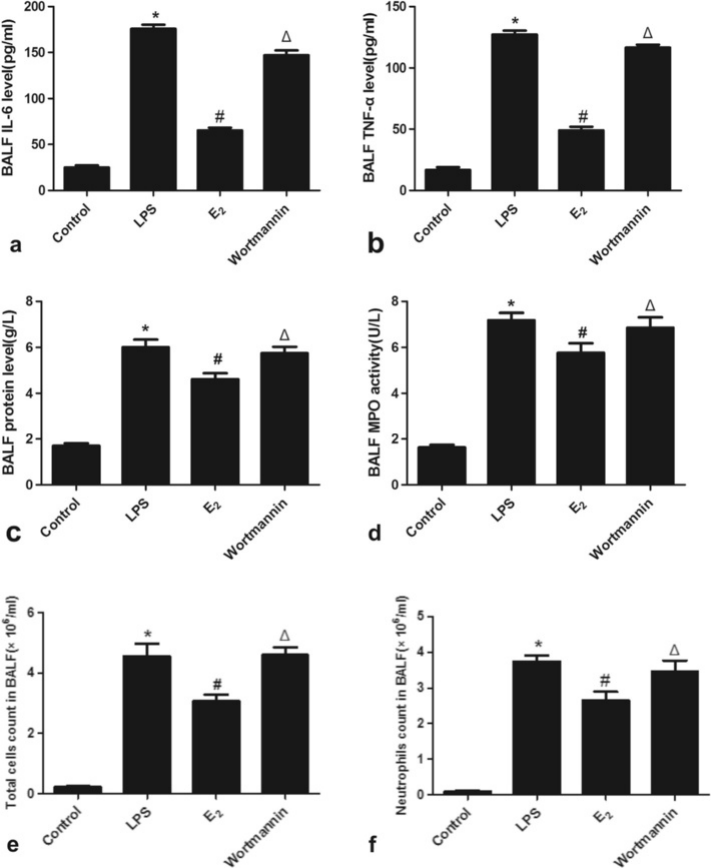
**Effects of 17β-estradiol (E**_**2**_ **) on the levels of IL-6 (a), TNF-α(b), protein levels (c), MPO activity (d), the total cell count(e), and the neutrophil count (f) in mouse BALF 4 hours after LPS treatment.** Data are presented as means ± S.E.M (*p < 0.05 compared with the control group, #p < 0.05 compared with the LPS group, Δp < 0.05 compared with the 17β-estradiol group).

To evaluate the effects of 17β-estradiol on LPS-induced inflammation, we analyzed TNF-α, IL-6, protein level, MPO activity and neutrophil infiltration in the BALF of treated mice. LPS significantly increased the levels of TNF-α and IL-6, and increased MPO activity and neutrophil counts. These effects were inhibited by 17β-estradiol in a PI3K dependent manner, as evidenced by the effects of wortmannin pre-treatment (Figure 2).

### 17β-estradiol ameliorated pulmonary edema and accelerated alveolar fluid clearance *in vivo*

**Figure 3:**
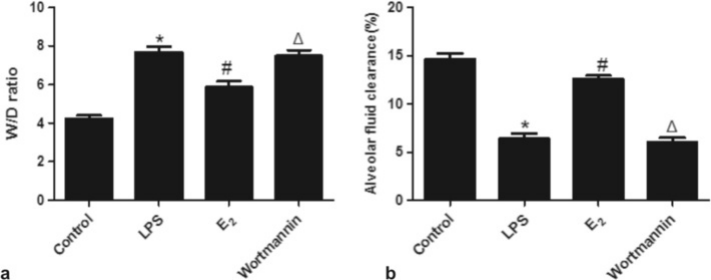
**Effects of 17β-estradiol (E**_**2**_ **) on W/D ratio (a) and alveolar fluid clearance (b) in mouse lungs 4 hours after LPS challenge.** Data are presented as means ± S.E.M (*p < 0.05 compared with the control group, #p < 0.05 compared with the LPS group, Δp < 0.05 compared with the 17β-estradiol group).

To assess pulmonary edema and the alveolar fluid removal efficiency in LPS treated mice, we calculated the lung W/D ratios and AFC rates of the mice. LPS significantly increased the W/D ratio, while the AFC rate was reduced by LPS, indicating induction of edema. However, edema was significantly reduced by 17β-estradiol treatment in a PI3K dependent manner. Pre-treatment of wortmannin also resulted in edema in the presence of 17β-estradiol, suggesting that 17β-estradiol acts through PI3K to prevent LPS-induced edema (Figure 3).

### 17β-estradiol did not affect mRNA transcription of α-, β-, γ-ENaC *in vivo* and *in vitro*

**Figure 4:**
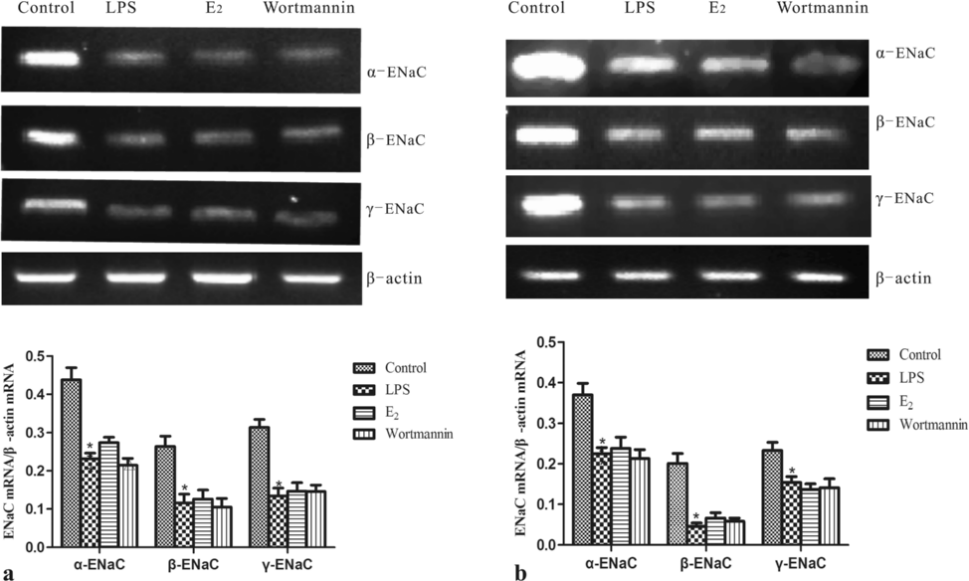
**Effects of 17β-estradiol (E**_**2**_ **) on the mRNA transcription levels of alveolar epithelial sodium channel (ENaC) in mouse lungs (a) and MLE-12 cells (b) after LPS treatment.** mRNA level was normalized to β-actin. Data are presented as means ± S.E.M (p < 0.05 compared with the control group. #p < 0.05 compared with the LPS group, Δp < 0.05 compared with the 17β-estradiol group).

To determine if 17β-estradiol affected the transcription of ENaC following LPS exposure, we measured ENaC mRNA levels in the lungs of treated mice and in LPS treated MLE-12 cells by reverse transcriptase PCR analysis. LPS exposure significantly reduced mRNA levels of the α-, β-, and γ- ENaC subunits. However, administration of 17β-estradiol did not affect the LPS-induced reduction of ENaC mRNA (Figure 4). These results suggest that 17β-estradiol does not affect ENaC transcription.

### 17β-estradiol up-regulated α-ENaC total protein expression *in vivo* and *in vitro*

**Figure 5:**
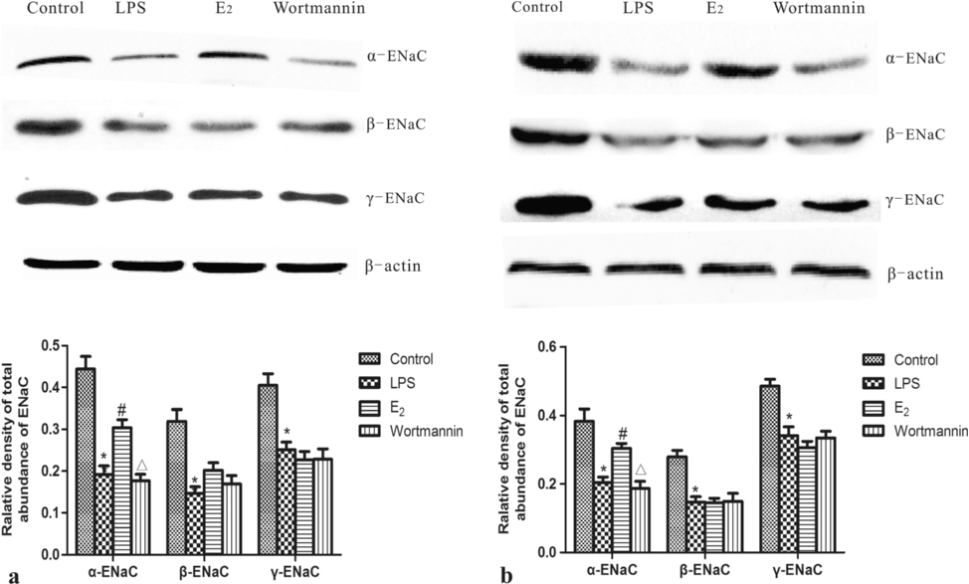
**Effects of 17β-estradiol (E**_**2**_ **) on total protein expression levels of alveolar epithelial sodium channel (ENaC) in mouse lungs (a) and MLE-12 cells (b) after LPS treatment.** Protein level was normalized to β-actin. Data are presented as means ± S.E.M (*p < 0.05 compared with the control group, #p < 0.05 compared with the LPS group, Δp < 0.05 compared with the 17β-estradiol group).

**Figure 6:**
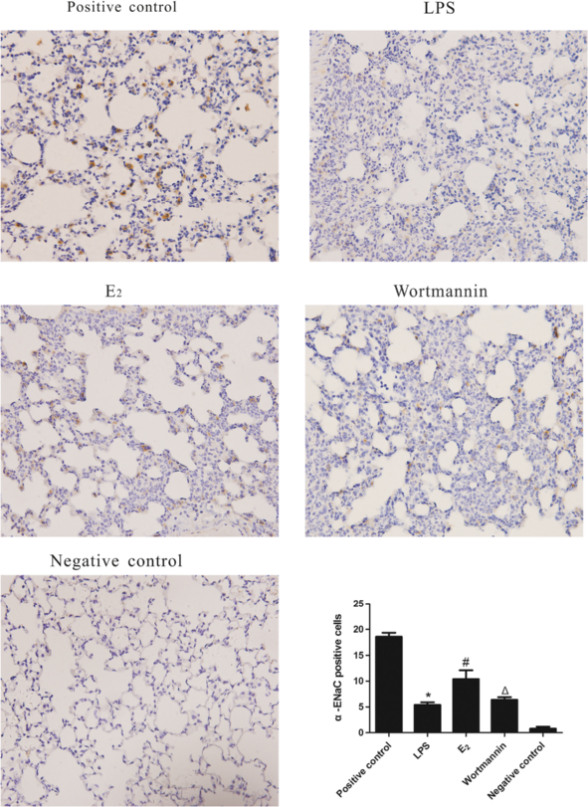
**Effects of 17β-estradiol (E**_**2**_ **) on the total protein expression levels of alveolar α**-**ENaC in mouse lungs after LPS treatment (immunohistochemistry stain, magnification × 400).** Data are presented as mean ± S.E.M (*p < 0.05 compared with the control group, #p < 0.05 compared with the LPS group, Δp < 0.05 compared with the 17β-estradiol group).

To determine if 17β-estradiol regulates the protein levels of ENaC, western blot analysis was employed to measure ENaC total protein levels in mouse lung tissues and MLE-12 cells, furthermore, immunohistochemistry analysis was employed to assess α-ENaC total protein levels in mouse lung tissues. Consistent with our mRNA analysis, we found that α-, β-, γ-ENaC total protein levels were down-regulated in LPS-treated lungs and cells. However, α-ENaC protein levels were significantly higher in 17β-estradiol treated mice and cells, but not in those pretreated with wortmannin (Figures 5 and 6). No significant differences were observed between the protein levels of β- ENaC and γ-ENaC. These results suggest that 17β-estradiol can stabilize α-ENaC protein levels through a PI3K-dependent mechanism.

### 17β-estradiol up-regulated α-ENaC membrane abundance *in vivo* and *in vitro*

**Figure 7:**
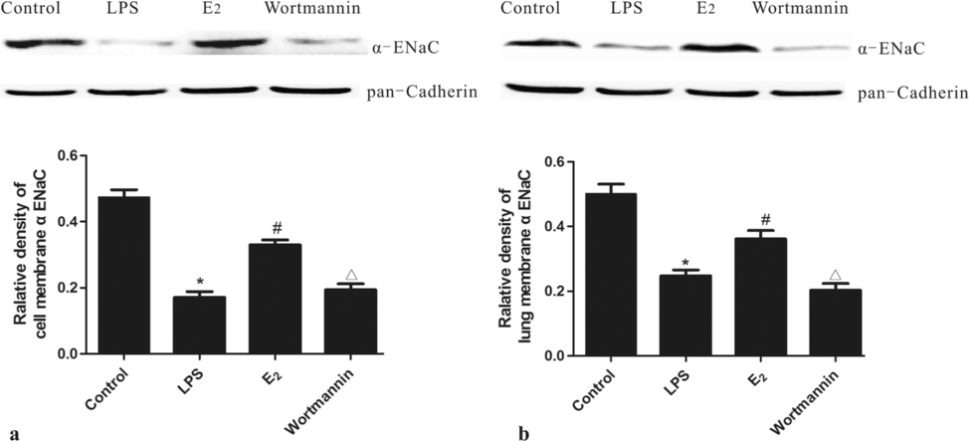
**Effects of 17β-estradiol (E**_**2**_ **) on the membrane abundance of α**-**ENaC protein in mouse lungs (a) and MLE-12 cells (b) after LPS treatment.** Membrane abundance of α-ENaC protein was normalized to pan-cadherin. Data are presented as mean ± S.E.M (*p < 0.05 compared with the control group, #p < 0.05 compared with the LPS group, Δp < 0.05 compared with the 17β-estradiol group).

In the next experiment, we further examined the membrane abundance of α-ENaC protein in the lung tissues and MLE-12 cells by western blot analysis. The membrane abundance of α-ENaC was reduced by LPS treatment, while this reduction was blocked by administration of 17β-estradiol *in vivo* and *in vitro*. Pre-treatment with wortmannin prevented the 17β-estradiol-induced up-regulation of α-ENaC (Figure 7). These results suggest that 17β-estradiol can promote α-ENaC membrane abundance in a PI3K-dependent manner.

### 17β-estradiol activated PI3K/Akt /SGK1 pathway *in vivo* and *in vitro*

**Figure 8:**
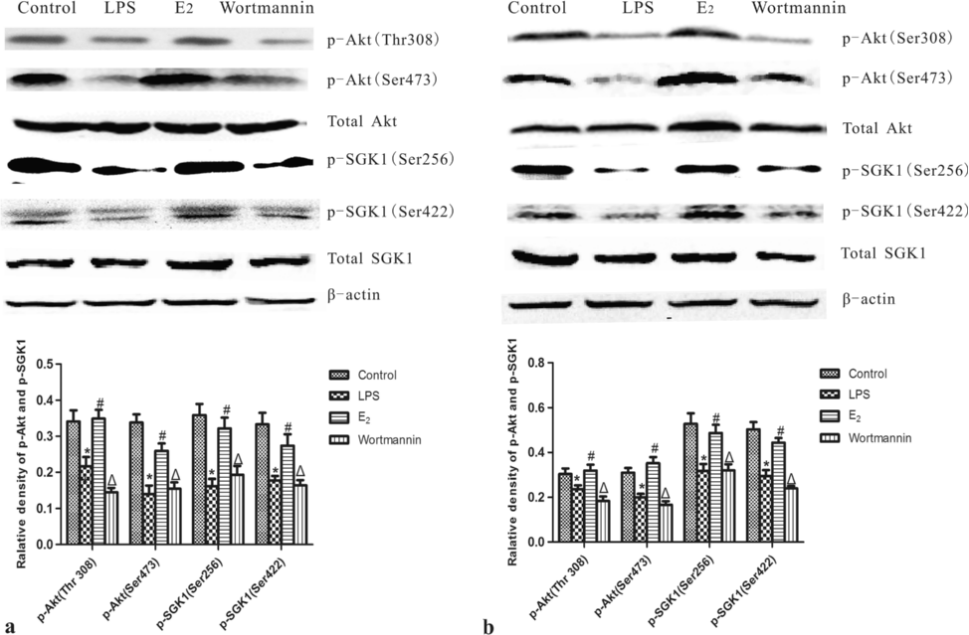
**Effects of 17β-estradiol (E**_**2**_ **) on the Akt and SGK1 phosphorylation levels in mouse lungs (a) and in whole cell lysates from MLE-12 cells (b) after LPS treatment.** Phosphorylation levels were normalized to β-actin. Data are presented as means ± S.E.M (*p < 0.05 compared with the control group, #p < 0.05 compared with the LPS group, Δp < 0.05 compared with the 17β-estradiol group).

As the therapeutic effects of 17β-estradiol on ALI appeared to be PI3K-dependent, we assessed the PI3K signaling pathway by western blot analysis. Compared to the lungs of control mice, phosphorylated Akt and SGK1 levels were significantly reduced in LPS treated mouse lungs. However, this reduction was blocked by administration of 17β-estradiol. Pre-treatment with wortmannin prevented the effects of 17β-estradiol. Similar results were observed in LPS-treated MLE-12 cells (Figure 8).

## Discussion

Our data suggest that 17β-estradiol plays a protective role in LPS-induced ALI. These data would be consistent with the reduced severity of ARDS/ALI commonly observed in female patients [3-8]. In our mouse model of LPS-induced ALI, 17β-estradiol attenuated lung histopathhologic damage, inflammatory response, and neutrophil infiltration in LPS-exposed lungs. Moreover, 17β-estradiol also reduced pulmonary edema and increased AFC rates following LPS exposure. As pre-treatment of mice with the PI3K inhibitor wortmannin prevented the protective effect of 17β-estradiol, it appears to act through PI3K to suppress ALI. Studies have demonstrated the sexual dimorphism on ENaC regulation in several tissues, including the lung tissue [20-23,42-45]. Consistent with these findings, we observed that 17β-estradiol stabilized total and surface levels of α-ENaC in LPS-treated lungs and MLE-12 cells in a PI3K-dependent manner. It is likely that the elevation of α-ENaC protein expression and membrane abundance contributes to the reduced edema and elevated AFC rates, thereby attenuating ALI following LPS exposure. Furthermore, our data indicate that 17β-estradiol reverses the LPS-induced reduction in Akt and SGK1 phosphorylation, which were abolished by the wortmannin, further suggesting that 17β-estradiol exerts its effects through PI3K. Collectively, our findings indicate that 17β-estradiol exerts beneficial effects at the early stage of ALI by repressing inflammatory responses and elevating α-ENaC protein expression and membrane abundance, at least partially through PI3K/Akt/SGK1 signaling pathway.

In most clinical research, female subjects have lower morbidity and mortality from trauma, ischemia/reperfusion, shock, and sepsis, which are the common risk factors of ARDS [3-8]. Moreover, animal models suggest that high 17β-estradiol levels, due to endogenous or exogenous administration, exert protective effects on attenuation of lung injury in a variety of settings [9-14].

ARDS/ALI usually develops in patients with a systemic inflammatory response, such as sepsis, major trauma, aspiration pneumonia and acute pancreatitis, among which severe sepsis is the most common risk factor for ARDS/ALI [2,46]. Clinical studies have found lower rates of sepsis and multi-organ failure following trauma haemorrhage in female subjects compare to males [47,48]. Moreover, postpubertal males exhibit higher sepsis mortality and greater severity of illness on PICU admission [49]. As a component of Gram-negative bacteria cellular walls, LPS exposure can induce sepsis and is a commonly used experimental model for ALI/ARDS [50,51]. Although experimental studies demonstrated a more severe LPS-induced ALI in male and ovariectomized female mice than intact female mice due to an anti-inflammatory effect of estrogen, other mechanisms underlying its protective effects are still under investigated [14,52]. Our data indicate that, consistent with other studies, 17β-estradiol has an anti-inflammatory effect in cases of LPS-associated ALI. Moreover this effect appears to occur through a PI3K-dependent mechanism. These results are consistent with previous studies demonstrating that PI3K/Akt signaling pathway plays a crucial role in the attenuation of inflammation as part of a negative feedback loop in response to injury [30,31].

Besides an inflammatory response, ARDS/ALI is characterized by proteinaceous pulmonary edema that floods the airspace and impedes gas exchange. Efficient alveolar fluid clearance (AFC), a process in which superfluous edema is removed by ion transporter, is associated with a positive outcome of ALI/ARDS [15,16], and alveolar epithelial sodium channel (ENaC) plays a rate-limiting role in the maintenance of AFC [17,18]. We observed that the beneficial effects of 17β-estradiol were associated with the up-regulation of α-ENaC protein and membrane abundance. These results are consistent with previous studies demonstrating higher AFC rates in females compared to males [19] and pro-absorptive functions of estrogen and progesterone in bronchial epithelium by regulating airway ENaC [20-23].

In addition, as another important regulator for sodium trans-epithelial absorption, Na/K-ATPases plays a synergistic role in AFC [53]. However, little is known about the effects of female hormones on this ion transporter. The combination of estradiol and progesterone can increase the ouabain-sensitive current and the mRNA expression of Na,K-ATPases β1 subunit, but does not alter the protein expression of Na,K-ATPases subunits [23]. Nevertheless, we focus on ENaC regulation for AFC in our present study. Much is yet to be investigated for the regulation and mechanism of ion channels by 17β-estradiol in pulmonary epithelium.

In our model, 17β-estradiol stabilized α-ENaC protein expression and membrane localization, but did not affect mRNA levels of it, suggesting that 17β-estradiol regulates α-ENaC through a non-genomic mechanism. Indeed, 17β-estradiol can regulate proteins expression and trafficking through both genomic and non-genomic mechanism [24,25]. 17β-estradiol was initially thought to exert its effects through a genomic mechanism to regulate protein synthesis, a process which takes hours to days. Recent research has highlighted the role of the non-genomic functions of 17β-estradiol, which includes the rapid activation of signaling pathways such as PI3K, ERK, and MAPK to modulate the expression, function, and distribution of proteins [12,24,25].

In particular, the PI3K pathway can activate serum and glucocorticoid-induced kinanse-1 (SGK1), a portent regulator of ENaC, by phosphorylation of PDK1 and mTORC2 [33-35]. SGK1 can up-regulate ENaC through phosphorylation of Nedd4-2, an E3 ubiquitin protein ligase, to inhibit α-ENaC degradation and subsequently increase α-ENaC membrane abundance and activity [54]. Recent findings indicate that SGK1 can promote ENaC trafficking to the cell membrane via phosphorylation of Rab11b [55], thereby increasing ENaC membrane abundance. Our results indicate that 17β-estradiol stabilizes α-ENaC protein expression and membrane abundance following LPS treatment. This likely occurs through increased membrane trafficking of ENaC as well as decreased degradation of ENaC via a SGK1-mediated mechanism. Furthermore, it is known that 17β-estradiol can activate the PI3K/Akt pathway to attenuate lung injury induced by trauma-hemorrhage and acute pancreatitis through non-genomic mechanisms [40,41]. However, the role of this signaling axis in LPS-induced ALI remains poorly defined. Our results suggest that PI3K-dependent activation of SGK1 can promote both the total expression and membrane abundance of α-ENaC, and contribute to a protective effect in cases of LPS-induced ALI.

In conclusion, our study demonstrates that 17β-estradiol can have a protective effect at the early stage of LPS-induced ALI by suppressing inflammation and pulmonary edema. These protective effects occur, at least in part, via the rapid non-genomic mechanism involving the activation of PI3K/Akt/SGK1 signaling pathway, ultimately resulting in stabilization of α-ENaC and the reduction of edema. Our results provide new insight into the mechanisms that underlie sexual dimorphism in ARDS, and may suggest novel therapeutic interventions for these patients. Further studies are necessary to define the signaling pathways that mediate the protective effects of 17β-estradiol and the specific and overlapping effects of 17β-estradiol-mediated genomic and non-genomic molecular actions.

## References

[1] Avecillas JF, Freire AX, Arroliga AC (2006). Clinical epidemiology of acute lung injury and acute respiratory distress syndrome: incidence, diagnosis, and outcomes. Clin Chest Med.

[2] Rubenfeld GD, Caldwell E, Peabody E, Weaver J, Martin DP, Neff M, Stern EJ, Hudson LD (2005). Incidence and outcomes of acute lung injury. N Engl J Med.

[3] Phua J, Badia JR, Adhikari NK, Friedrich JO, Fowler RA, Singh JM, Scales DC, Stather DR, Li A, Jones A, Gattas DJ, Hallett D, Tomlinson G, Stewart TE, Ferguson ND (2009). Has mortality from acute respiratory distress syndrome decreased over time?: A systematic review. Am J Respir Crit Care Med.

[4] Cardinal-Fernández P, Ferruelo A, El-Assar M, Santiago C, Gómez-Gallego F, Martín-Pellicer A, Frutos-Vivar F, Peñuelas O, Nin N, Esteban A, Lorente JA (2013). Genetic predisposition to acute respiratory distress syndrome in patients with severe sepsis. Shock.

[5] Anadkat JS, Kuzniewicz MW, Chaudhari BP, Cole FS, Hamvas A (2012). Increased risk for respiratory distress among white, male, late preterm and term infants. J Perinatol.

[6] Agarwal R, Aggarwal AN, Gupta D, Behera D, Jindal SK (2006). Etiology and outcomes of pulmonary and extrapulmonary acute lung injury/ARDS in a respiratory ICU in North India. Chest.

[7] Moss M, Mannino DM (2002). Race and gender differences in acute respiratory distress syndrome deaths in the United States: an analysis of multiple-cause mortality data (1979–1996). Crit Care Med.

[8] Lahm T, Crisostomo PR, Markel TA, Wang M, Weil BR, Novotny NM, Meldrum DR (2008). The effects of estrogen on pulmonary artery vasoreactivity and hypoxic pulmonary vasoconstriction: potential new clinical implications for an old hormone. Crit Care Med.

[9] Hamidi SA, Dickman KG, Berisha H, Said SI: **17β-estradiol protects the lung against acute injury: possible mediation by vasoactive intestinal polypeptide.** *Endocrinology* 2011, **152:**4729–37. 10.1210/en.2011-1631 PMC3230060 22009726

[10] Cuzzocrea S, Mazzon E, Sautebin L, Serraino I, Dugo L, Calabró G, Caputi AP, Maggi A (2001). The protective role of endogenous estrogens in carrageenan-induced lung injury in the rat. Mol Med.

[11] Fan Q, Zhao P, Li J, Xie X, Xu M, Zhang Y, Mu D, Li W, Sun R, Liu W, Nan Y, Zhang B, Jin F, Li Z (2011). 17β-Estradiol administration attenuates seawater aspiration-induced acute lung injury in rats. Pulm Pharmacol Ther.

[12] Hsu JT, Kan WH, Hsieh CH, Choudhry MA, Bland KI, Chaudry IH (2009). Role of extracellular signal-regulated protein kinase (ERK) in 17beta-estradiol-mediated attenuation of lung injury after trauma-hemorrhage. Surgery.

[13] Breithaupt-Faloppa AC, Fantozzi ET, de Assis Ramos MM, Vitoretti LB, Couto GK, Lino-dos-Santos-Franco A, Rossoni LV, Oliveira-Filho RM, Vargaftig BB, Tavares-de-Lima W (2013). Protective effect of estradiol on acute lung inflammation induced by an intestinal ischemic insult is dependent on nitric oxide. Shock.

[14] Speyer CL, Rancilio NJ, McClintock SD, Crawford JD, Gao H, Sarma JV, Ward PA (2005). Regulatory effects of estrogen on acute lung inflammation in mice. Am J Physiol Cell Physiol.

[15] Ware LB, Matthay MA (2001). Alveolar fluid clearance is impaired in the majority of patients with acute lung injury and the acute respiratory distress syndrome. Am J Respir Crit Care Med.

[16] Morty RE, Eickelberg O, Seeger W (2007). Alveolar fluid clearance in acute lung injury: what have we learned from animal models and clinical studies?. Intensive Care Med.

[17] Matthay MA, Folkesson HG, Clerici C (2002). Lung epithelial fluid transport and the resolution of pulmonary edema. Physiol Rev.

[18] Hummler E, Planès C (2010). Importance of ENaC-mediated sodium transport in alveolar fluid clearance using genetically-engineered mice. Cell Physiol Biochem.

[19] Bastarache JA, Ong T, Matthay MA, Ware LB (2011). Alveolar fluid clearance is faster in women with acute lung injury compared to men. J Crit Care.

[20] Sweezey N, Tchepichev S, Gagnon S, Fertuck K, O’Brodovich H (1998). Female gender hormones regulate mRNA levels and function of the rat lung epithelial Na channel. Am J Physiol Cell Physiol.

[21] Greenlee MM, Mitzelfelt JD, Yu L, Yue Q, Duke BJ, Harrell CS, Neigh GN, Eaton DC (2013). Estradiol activates epithelial sodium channels in rat alveolar cells through the G protein-coupled estrogen receptor. Am J Physiol Lung Cell Mol Physiol.

[22] Trotter A, Ebsen M, Kiossis E, Meggle S, Kueppers E, Beyer C, Pohlandt F, Maier L, Thome UH (2006). Prenatal estrogen and progesterone deprivation impairs alveolar formation and fluid clearance of newborn piglets. Pediatr Res.

[23] Laube M, Kuppers E, Thome UH (2011). Modulation of sodium transport in alveolar epithelial cells by estradiol and progesterone. Pediatr Res.

[24] Björnström L, Sjöberg M (2005). Mechanisms of estrogen receptor signaling: convergence of genomic and nongenomic actions on target genes. Mol Endocrinol.

[25] Ho KJ, Liao JK (2002). Nonnuclear actions of estrogen. Arterioscler Thromb Vasc Biol.

[26] Yu HP, Hsieh YC, Suzuki T, Choudhry MA, Schwacha MG, Bland KI, Chaudry IH (2007). The PI3K/Akt pathway mediates the nongenomic cardioprotective effects of estrogen following trauma-hemorrhage. Ann Surg.

[27] Hsu JT, Kan WH, Hsieh CH, Choudhry MA, Schwacha MG, Bland KI, Chaudry IH (2007). Mechanism of estrogen mediated attenuation of hepatic injury following traumahemorrhage: Akt-dependent HO-1 up-regulation. J Leukoc Biol.

[28] Hisamoto K, Ohmichi M, Kurachi H, Hayakawa J, Kanda Y, Nishio Y, Adachi K, Tasaka K, Miyoshi E, Fujiwara N, Taniguchi N, Murata Y (2001). Estrogen induces the Akt-dependent activation of endothelial nitric-oxide synthase in vascular endothelial cells. J Biol Chem.

[29] Honda K, Sawada H, Kihara T, Urushitani M, Nakamizo T, Akaike A, Shimohama S (2000). Phosphatidylinositol 3-kinase mediates neuroprotection by estrogen in cultured cortical neurons. J Neurosci Res.

[30] Williams DL, Ozment-Skelton T, Li C (2006). Modulation of the phosphoinositide 3-kinase signaling pathway alters host response to sepsis, inflammation, and ischemia/reperfusion injury. Shock.

[31] Schabbauer G, Tencati M, Pedersen B, Pawlinski R, Mackman N (2004). PI3K-Akt pathway suppresses coagulation and inflammation in endotoxemic mice. Arterioscler Thromb Vasc Biol.

[32] Manukyan MC, Weil BR, Wang Y, Abarbanell AM, Herrmann JL, Poynter JA, Meldrum DR (2010). The phosphoinositide-3 kinase survival signaling mechanism in sepsis. Shock.

[33] Baines D (2013). Kinases as targets for ENaC regulation. Curr Mol Pharmacol.

[34] Lang F, Artunc F, Vallon V (2009). The physiological impact of the serum and glucocorticoid-inducible kinase SGK1. Curr Opin Nephrol Hypertens.

[35] Lang F, Bohmer C, Palmada M, Seebohm G, Strutz-Seebohm N, Vallon V (2006). (Patho)physiological significance of the serum- and glucocorticoid-inducible kinase isoforms. Physiol Rev.

[36] Kolliputi N, Waxman AB (2009). IL-6 cytoprotection in hyperoxic acute lung injury occurs via PI3K/Akt-mediated Bax phosphorylation. Am J Physiol Lung Cell Mol Physiol.

[37] Deng W, Li CY, Tong J, Zhang W, Wang DX (2012). Regulation of ENaC-mediated alveolar fluid clearance by insulin via PI3K/Akt pathway in LPS-induced acute lung injury. Respir Res.

[38] Lee JP, Li YC, Chen HY, Lin RH, Huang SS, Chen HL, Kuan PC, Liao MF, Chen CJ, Kuan YH (2010). Protective effects of luteolin against lipopolysaccharide-induced acute lung injury involves inhibition of MEK/ERK and PI3K/Akt pathways in neutrophils. Acta Pharmacol Sin.

[39] Peng XQ, Damarla M, Skirball J, Nonas S, Wang XY, Han EJ, Hasan EJ, Cao X, Boueiz A, Damico R, Tuder RM, Sciuto AM, Anderson DR, Garcia JG, Kass DA, Hassoun PM, Zhang JT (2010). Protective role of PI3-kinase/Akt/eNOS signaling in mechanical stress through inhibition of p38 mitogen-activated protein kinase in mouse lung. Acta Pharmacol Sin.

[40] Yang SJ, Chen HM, Hsieh CH, Hsu JT, Yeh CN, Yeh TS, Hwang TL, Jan YY, Chen MF (2011). Akt pathway is required for oestrogen-mediated attenuation of lung injury in a rodent model of cerulein-induced acute pancreatitis. Injury.

[41] Hsu JT, Yeh HC, Chen TH, Kuo CJ, Lin CJ, Chiang KC, Yeh TS, Hwang TL, Chaudry II (2013). Role of Akt/HO-1 pathway in estrogen-mediated attenuation of trauma-hemorrhage-induced lung injury. J Surg Res.

[42] Saint-Criq V, Rapetti-Mauss R, Yusef YR, Harvey BJ (2012). Estrogen regulation of epithelial ion transport: Implications in health and disease. Steroids.

[43] Yang GZ, Nie HG, Lu L, Chen J, Lu XY, Ji HL, Li QN (2011). Estrogen regulates the expression and activity of epithelial sodium channel in mouse osteoblasts. Cell Mol Biol (Noisy-le-grand).

[44] Chang CT, Sun CY, Pong CY, Chen YC, Lin GP, Chang TC, Wu MS (2007). Interaction of estrogen and progesterone in the regulation of sodium channels in collecting tubular cells. Chang Gung Med J.

[45] Heo NJ, Son MJ, Lee JW, Jung JY, Kim S, Oh YK, Na KY, Yoon HJ, Joo KW, Han JS (2013). Effect of estradiol on the expression of renal sodium transporters in rats. Climacteric.

[46] Sheu CC, Gong MN, Zhai R, Chen F, Bajwa EK, Clardy PF, Gallagher DC, Thompson BT, Christiani DC (2010). Clinical characteristics and outcomes of sepsis-related vs non-sepsis-related ARDS. Chest.

[47] Trentzsch H, Nienaber U, Behnke M, Lefering R, Piltz S (2014). Female sex protects from organ failure and sepsis after major trauma haemorrhage. Injury.

[48] Schoeneberg C, Kauther MD, Hussmann B, Keitel J, Schmitz D, Lendemans S (2013). Gender-specific differences in severely injured patients between 2002 and 2011: data analysis with matched-pair analysis. Crit Care.

[49] Ghuman AK, Newth CJ, Khemani RG (2013). Impact of gender on sepsis mortality and severity of illness for prepubertal and postpubertal children. J Pediatr.

[50] Ronco C (2014). Lipopolysaccharide (LPS) from the cellular wall of Gram-negative bacteria, also known as endotoxin, is a key molecule in the pathogenesis of sepsis and septic shock. Preface. Blood Purif.

[51] Rosenthal C, Caronia C, Quinn C, Lugo N, Sagy M (1998). A comparison among animal models of acute lung injury. Crit Care Med.

[52] Erikoglu M, Sahin M, Ozer S, Avunduk MC (2005). Effects of gender on the severity of sepsis. Surg Today.

[53] Looney MR, Sartori C, Chakraborty S, James PF, Lingrel JB, Matthay MA (2005). Decreased expression of both the alpha1- and alpha2-subunits of the Na-K-ATPase reduces maximal alveolar epithelial fluid clearance. Am J Physiol Lung Cell Mol Physiol.

[54] Chandran S, Li H, Dong W, Krasinska K, Adams C, Alexandrova L, Chien A, Hallows KR, Bhalla V (2011). Neural precursor cell-expressed developmentally down-regulated protein 4–2 (Nedd4-2) regulation by 14-3-3 protein binding at canonical serum and glucocorticoid kinase 1 (SGK1) phosphorylation sites. J Biol Chem.

[55] Butterworth MB, Edinger RS, Silvis MR, Gallo LI, Liang X, Apodaca G, Frizzell RA, Johnson JP (2012). Rab11b regulates the trafficking and recycling of the epithelial sodium channel (ENaC). Am J Physiol Renal Physiol.

